# Human amniotic membrane for myocutaneous dehiscence after a radical surgical treatment of vulvar cancer: A case report

**DOI:** 10.3389/fonc.2022.1009884

**Published:** 2022-09-23

**Authors:** Stefano Restaino, Federico Paparcura, Cristina Giorgiutti, Diletta Trojan, Giulia Montagner, Giancarlo Pengo, Grazia Pividore, Roberta Albanese, Emanuele Rampino, Teresa Dogareschi, Tiziana Bove, Francesca Titone, Marco Trovò, Giorgia Garganese, Pier Camillo Parodi, Giovanni Scambia, Lorenza Driul, Giuseppe Vizzielli

**Affiliations:** ^1^ Department of Maternal and Child Health, Obstetrics and Gynecology Clinic, University Hospital of Udine, Udine, Italy; ^2^ Medical Area Department (Dipartimento di Area Medica, DAME), Università degli Studi di Udine, Udine, Italy; ^3^ Fondazione Banca dei tessuti di Treviso Onlus, Treviso, Italy; ^4^ Surgical Video Production & Multimedia Medical – Azienda Sanitaria Universitaria Friuli Centrale, Udine, Italy; ^5^ Piwimed s.r.l., Udine, Italy; ^6^ Dipartimento di Scienze Chirurgiche, Clinica di Chirurgia Plastica Ricostruttiva, Università degli Studi di Udine, Udine, Italy; ^7^ Department of Anesthesia and Intensive Care, University Hospital of Udine, Udine, Italy; ^8^ Radiation Oncology Department, University Hospital of Udine, Udine, Italy; ^9^ Gynecology and Breast Care Center, Mater Olbia Hospital, Olbia, Italy; ^10^ Dipartimento di Scienze della Vita e Sanità Pubblica, Università Cattolica del Sacro Cuore, Roma, Italy; ^11^ Dipartimento per la salute della Donna e del Bambino e della Salute Pubblica, Fondazione Policlinico Universitario Agostino Gemelli IRCCS, Unità Operativa Complessa Ginecologia Oncologica, Roma, Italy

**Keywords:** amniotic membrane, case report, allograft, vulvar cancer, dehiscence

## Abstract

**Background:**

The application of the amniotic membrane could have a favourable effect on tissue repair and regeneration. We report the first case of implant of an amniotic membrane in a patient affected by myo-cutaneous dehiscence, after a radical surgical treatment for vulvar cancer.

**Methods:**

We describe a case of a 74-years-old patient affected by vulvar cancer. After radiotherapy, the patient underwent to an anterior pelvic exenteration with uretero-ileo-cutaneostomy by Wallace, bilateral pelvic lymphadenectomy, omental biopsies, omental flap, bilateral inguinal lymphadenectomy, resection of ulcerated left inguinal lesion, reconstruction with left gracilis muscle flap and locoregional V-Y advancement flap. The patient developed a myo-cutaneous dehiscence. Two months after the surgery, following an accurate curettage of the wound and negative pressure therapy, a patch of human amniotic membrane was implanted.

**Results:**

The surgical procedure was easy, feasible and did not require long operating room times. No intraoperative or postoperative complications occurred. The results obtained were encouraging with a marked improvement in the surgical wound.

**Conclusion:**

the use of amniotic membranes was safely and easily performed to promote the healing of complicated surgical wounds.

## Introduction

Vulvar cancer is one of most rare gynecological tumors, in fact, it accounts for only 2-5% of the cases (1). Squamous cell carcinoma (SCC) of the vulva, the most common subtype, has traditionally been considered a disease of postmenopausal women, although the average age of incidence has decreased in recent years due to the increase in HPV infections worldwide (1–4). The treatment of vulvar cancer depends mainly on histology and staging (1, 2), other variables influencing the management are age, coexistence of comorbidities and the patient’s performance status. Treatment is predominantly surgical, particularly for SCC, although concomitant chemoradiation is an effective alternative, particularly for advanced tumors and those where pelvic exenteration would be necessary to obtain adequate surgical edges (1–5). The most severe and common postoperative complications of vulvar cancer surgery are lymphedema, lymphocele, and wound dehiscence. In particular, en bloc surgery raised complications and wound dehiscence to 70-90% of cases (6, 7). The use of negative pressure therapy is an option for the conservative management of large wounds dehiscence (8). Human amniotic membrane (HAM) has been reported as a versatile graft in many surgical interventions. The first clinical application of HAM dates back to 1910 (9) and since then several studies reports its efficacy, especially as a biological dressing for chronic wounds (10, 11), but also in gynecological surgery (12, 13). In fact, HAM promotes cell migration and proliferation; it has anti-inflammatory and antimicrobial properties without prompting immunoreaction in the recipient (14–17). We describe the first case of implantation of HAM in a patient affected by vulvar cancer with a myocutaneuos dehiscence.

## Case description

We report the case of a 74-year-old female patient with previous bilateral salpingo-oophorectomy and hysterectomy for uterine fibromatosis. She had a clinical history of hypertension and dyslipidaemia and a silent family history. The patient came to our attention due to the development of itching and vulvar oedema associated with leucoxantorrhoea. A gynaecological examination under narcosis with multiple biopsies was performed. Histological examination diagnosed unrelated HPV infiltrating squamous cell carcinoma cT3 N2 MX. Staging MRI and PET-CT were subsequently performed, which confirmed vulvar neoplasia with locoregional infiltration and bilateral lymph node localisation. Imaging also revealed the presence of a lung abdensation, which was biopsied and diagnosed as adenocarcinoma with pulmonary primitivity. The lung tumour underwent radiotherapy treatment, with complete response. On the subsequent PET-CT scan it was no longer described.

**Figure 1:**
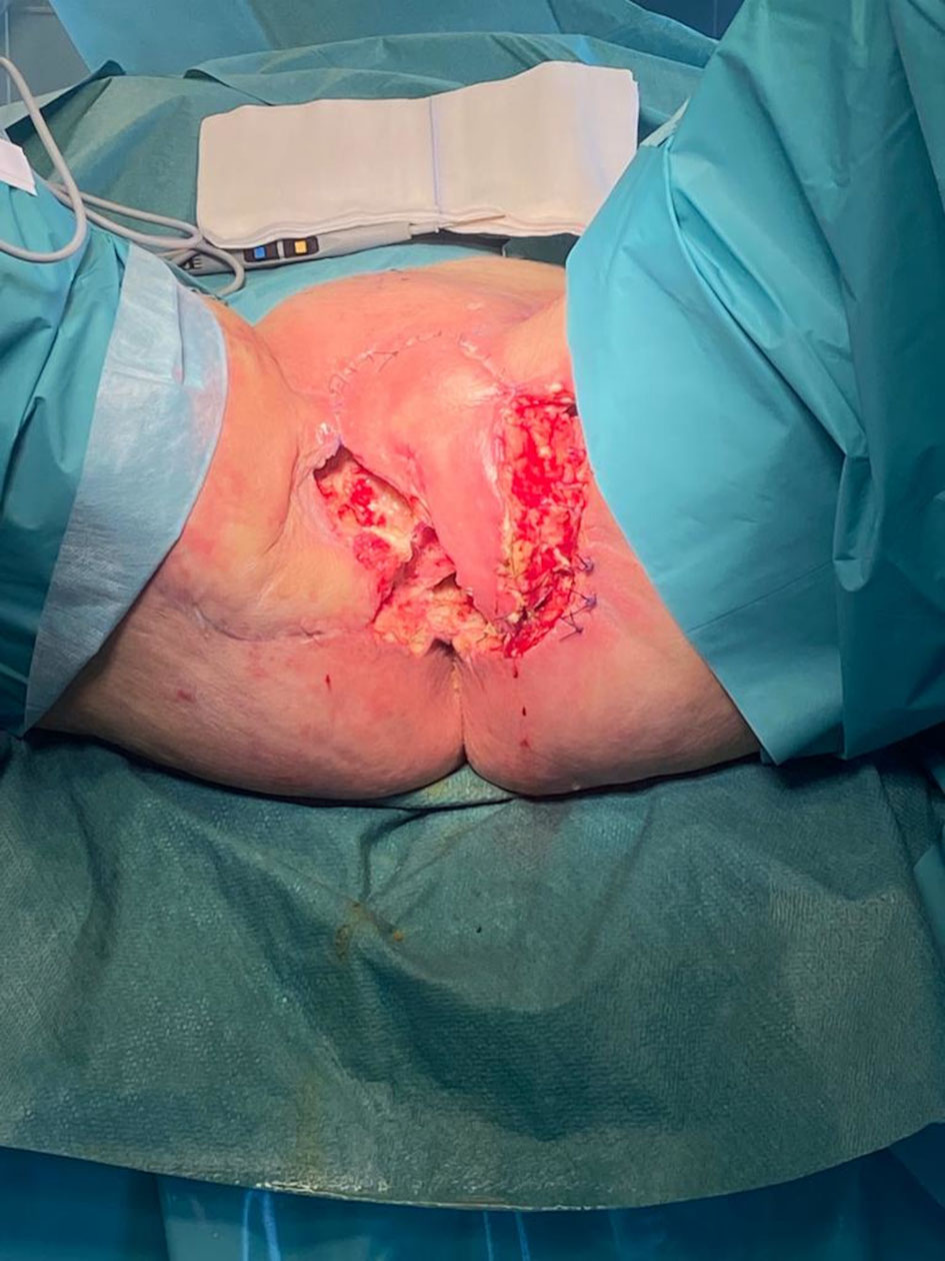
Myo-cutaneous dehiscence.

**Figure 2:**
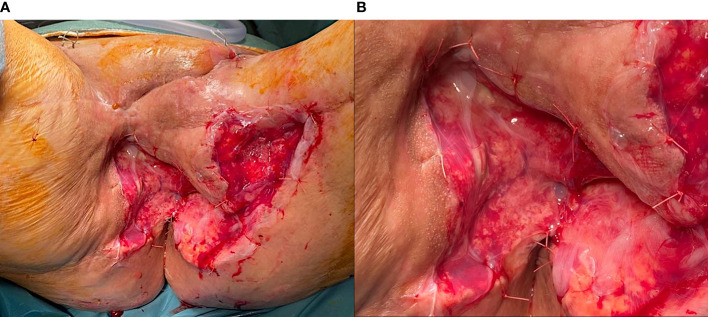
**(A)** Human amniotic membrane application. **(B)** Human amniotic membrane detail.

For the vulvar neoplasm, the patient underwent radiotherapy treatment for a total of 35 sessions. From 13^th^ July 2021 to 28^th^ September 2021, we performed exclusive radiotherapy treatment of vulvar neoplasm and loco-regional lymph node drainage (total dose 54 Gy in 35 sessions). Three months after radiotherapy treatment, we observed a persistent of the disease confirmed also by PET-CT scan examination. For this reason, the patient was submitted on 27^th^ January 2022 to an anterior pelvic exenteration with uretero-ileo-cutaneostomy packing by Wallace (18), bilateral pelvic lymphadenectomy, omental biopsies, omental flap, bilateral inguinal lymphadenectomy, resection of ulcerated left inguinal lesion, reconstruction with left gracilis muscle flap and locoregional V-Y advancement flap. Two days after surgery, the gracilis muscle flap was distressed with subsequent dehiscence of the surgical wound (**Figure 1**). For this reason, we decided to apply negative pressure therapy at the dehiscence site. Considering the difficulties in healing on 11^th^ April 2022 we decided to implant amniotic membrane on surgical wound because of its anti-inflammatory and antimicrobial properties (**Figures 2A, B**). The human amniotic membrane was provided by Treviso Tissue Bank Foundation, a non-profit health organization accredited by the National Transplant Centre. The placenta is collected from donors undergoing caesarean delivery, after their consent for the donation. Donors areselected and screened according to Italian requirement, that includes serological and molecular tests. The placenta is processed in a Good Manufacturing Practice (GMP) compliant facility, shortly after retrieval. The amniotic membrane is carefully detached from the chorion and rinsed with sterile saline solution to remove residual blood. Subsequently, amniotic membrane is immersed in a cocktail of antibiotics validated for tissue decontamination (19, 20). After the removal of blood and spongy residues, amniotic membrane is cut in patches of desired sizes, that are positioned on filters in contact with the stromal side, to keep the orientation. Amniotic membrane is then stored in vapor phase liquid nitrogen, immersed in a cryopreserving solution made up with Base medium (Alchimia Srl, Italy), DMSO (WAK-Chemie, Germany) and human albumin (Kedrion, Italy). On that date, the patient still had a continuous solution between the myocutaneous gracilis flap and the root of the right thigh, about 5-6 cm in longitudinal diameter, which deepened by about 2 cm. There was also a further continuous solution at the level of the root of the left thigh, the myocutaneous flap and the inguinal root, approximately 3 cm in maximum diameter and 1-2 cm deep, which was in continuity with the previous one at the level of the anal margin.

## Surgical procedure

**Figure 3:**
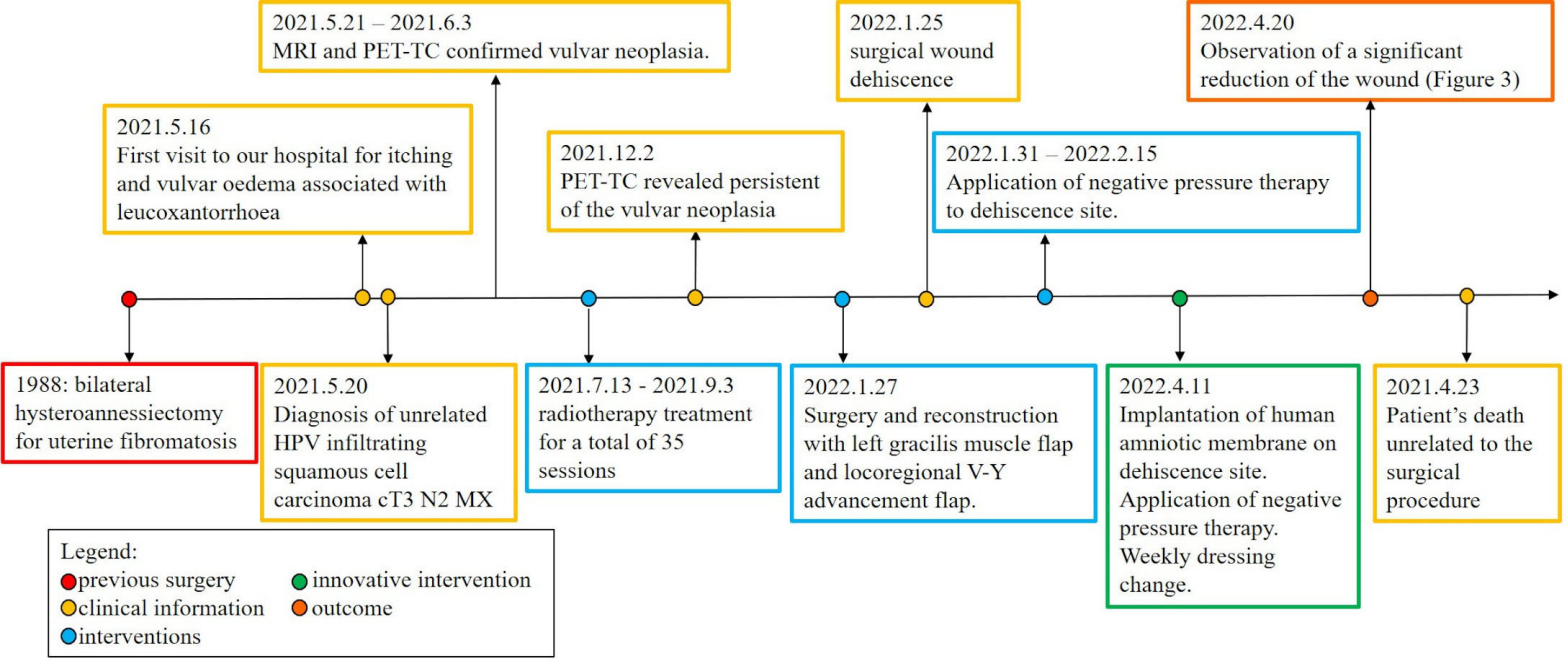
Timeline of the clinical case.

Under general anaesthesia, the patient was placed in the lithotomy position. We removed the negative pressure therapy. Following an accurate curettage of the vulvar scar with a curette to remove the granulation tissue, the tract was prepared for the implant of the HAM. The cryopreserved HAM resting on a filter was thawed in the operating room by immersion in a bath of saline solution at 40°C, without removing the packaging. It was subsequently washed twice in saline solution at 25°C; finally, one side of the square HAM was transfixed with a resorbable suture (2-0 Vicryl™, Ethicon Endo-Surgery, Inc., Cincinnati, OH, USA). Attention was paid to place the epithelial side of the HAM outward, to face the dehiscence wound (**Figures 2A, B**). The temporal timeline of the clinical case is showed in **Figure 3**.

## Results

**Figure 4:**
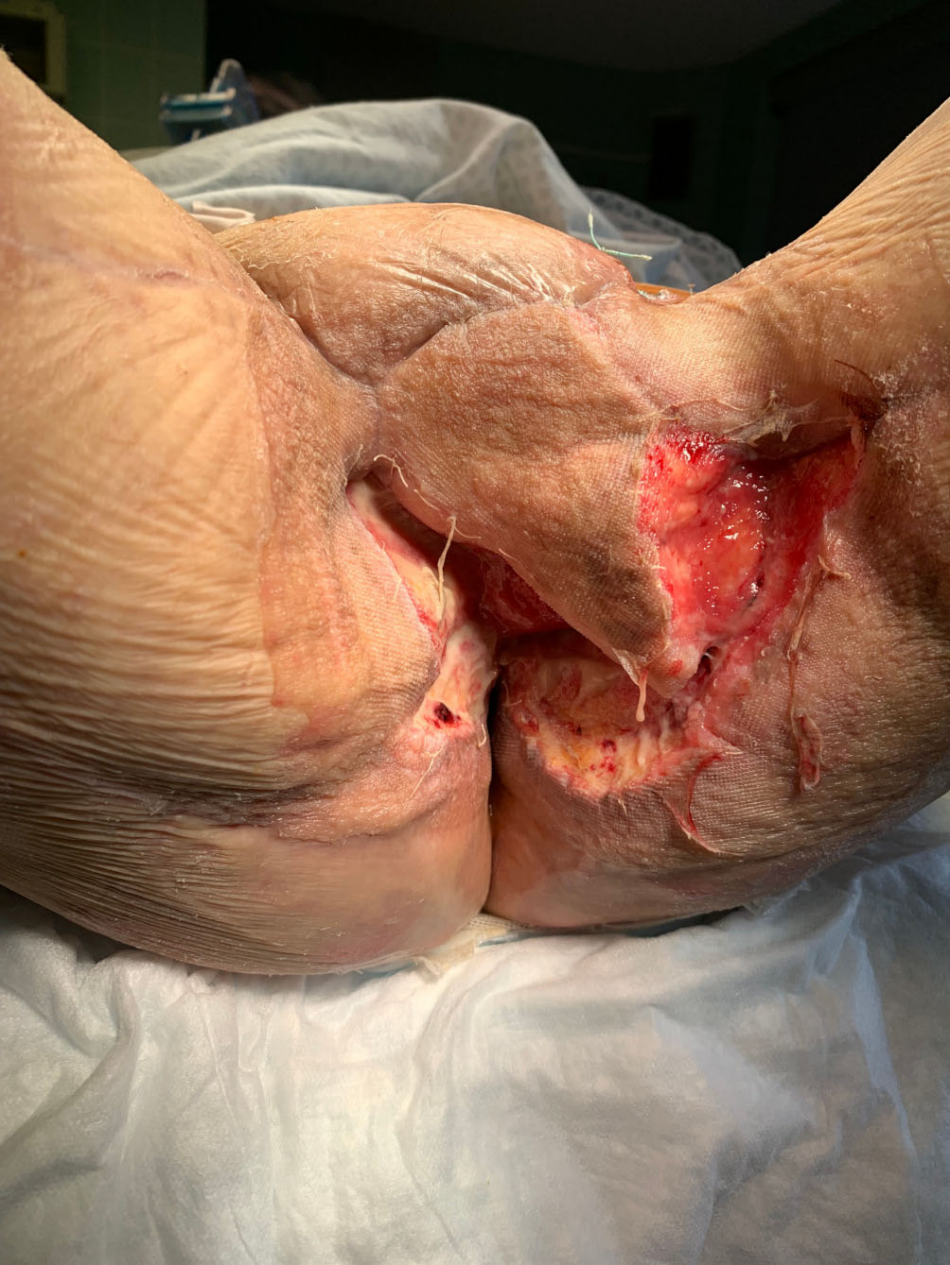
Effect of human amniotic membrane after two weeks.

No intraoperative or postoperative complications occurred. Stool softeners and analgesics were prescribed as needed. We continued to use negative pressure therapy at the wound site, changing the dressing weekly. Within 10 days of the amniotic membranes being placed, the wounds had significantly reduced (**Figure 4**). On 23^rd^ April 2022 the patient died for other circumstances unrelated to the surgical procedure. However, the results obtained were encouraging with a marked improvement in the surgical wound.

## Discussion

The implantation of amniotic membranes on surgical wounds appears to be safe; moreover, the psychological impact of the treatment on our patient was acceptable, with an improvement also in terms of pain and thus quality of life. HAM has already been used in several sites of the gastrointestinal tract, such as the duodenum, colon and rectovaginal fistula (21, 22). The use of HAM is also a widespread clinical practice in other fields of medicine such as eye surgery and the treatment of an increasing number of ocular surface diseases (23).

To our knowledge, this is the first case of using HAM to promote healing of a surgical wound in a patient with gynecological oncology. The benefits of amniotic membrane placement in difficult-to-heal surgical wounds could be related to its anti-inflammatory and antimicrobial properties and low immunogenicity (14–17). In fact, no immunosuppression therapy was administered to the patients and no immune reaction was triggered by the HAM, analogously to other published clinical application of this graft (24–27).

Due to sudden death, it was not possible to evaluate the long-term outcome of this procedure, but the results obtained seemed encouraging, considering the progression of healing achieved in the first two weeks after application of the amniotic membrane.

-.1This is an isolated case of the application of HAM, with a follow-up that is too limited to establish their real effectiveness.

Within the limits of this case report, a promising suggestion has been made regarding the use of HAM for gynecological surgery.

## Conclusion

Taking into account the high risk of dehiscence in vulvar cancer patients undergoing radical surgery, the use of HAMs could be a weapon to be considered. Case series providing longer follow-up should be encouraged, in order to provide useful data for increased use of this technique. In particular, future clinical studies could aim at comparing the efficacy of HAM versus traditional techniques.

## Data availability statement

The raw data supporting the conclusions of this article will be made available by the authors, without undue reservation.

## Ethics statement

Ethical review and approval was not required for the study on human participants in accordance with the local legislation and institutional requirements. The patients/participants provided their written informed consent to participate in this study. Written informed consent was obtained from the individual(s) for the publication of any potentially identifiable images or data included in this article.

## Author contributions

SR: Conceptualization and original draft preparation; FP: data curation and methodology; CG: data curation; GM and DT were involved in the drafting and revision of the manuscript. GP: software; GP: visualization; RA: supervision; ER: supervision; TD: methodology; TB: supervision; GG: validation; PP: validation; GS: validation; LD: validation; GV: Conceptualization and supervision. All authors contributed to the article and approved the submitted version.

## Acknowledgments

We thank Dr Morandini Marzia, Dr Barbui Elisa, Dr Spampinato Angelina, all nursing personnel and operating room staff of the Obstetrics and Gynecology Unit.

## Conflict of interest

Author GPi was employed by Piwimed s.r.l.

The remaining authors declare that the research was conducted in the absence of any commercial or financial relationships that could be construed as a potential conflict of interest.

## Publisher’s note

All claims expressed in this article are solely those of the authors and do not necessarily represent those of their affiliated organizations, or those of the publisher, the editors and the reviewers. Any product that may be evaluated in this article, or claim that may be made by its manufacturer, is not guaranteed or endorsed by the publisher.
